# Reoralisierung nach Intubation bei Intensivaufenthalt: Von der Nasensonde zum Butterbrot – ein Fallbericht

**DOI:** 10.1007/s00739-022-00833-5

**Published:** 2022-08-22

**Authors:** Martina Plementas, Michaela Trapl-Grundschober

**Affiliations:** 1 Klinische Abteilung für Neurologie, Universitätsklinikum Tulln, Karl Landsteiner Privatuniversität für Gesundheitswissenschaften Tulln, Tulln an der Donau, Österreich; 2 Klinische Abteilung für Neurologie, Therapeutischer Dienst, Universitätsklinikum Tulln, Karl Landsteiner Privatuniversität für Gesundheitswissenschaften, Alter Ziegelweg 10, 3430 Tulln an der Donau, Österreich

**Keywords:** Dysphagie, Lebensqualität, Autismus, Logopädie, Gugging Swallowing Screen, Dysphagia, Quality of life, Autism, Speech and language therapy and dysphagiology, Gugging Swallowing Screen

## Abstract

Die Schluckfunktion kann durch eine Intubation stark beeinträchtigt werden. Essen und Trinken, aber auch Speichelschlucken können dadurch massiv gestört werden. In diesem Fall konnten einer jungen, multimorbiden Frau durch die Zusammenarbeit des gesamten medizinischen Personals die Nahrungsaufnahme wieder ermöglicht und die Lebensqualität gesteigert werden.

## Abstract (en)

Swallowing function can be severely impaired by intubation. As a result, eating, drinking and swallowing saliva can also be tremendously affected. In this case, a young, multimorbid woman was able to regain the ability to eat and drink with the help of all medical staff, which significantly improved her quality of life.

## Einleitung

Essen und trinken zu können, deckt nicht nur den Kalorien- und Flüssigkeitsbedarf. Es bedeutet auch Lebensqualität, Zuwendung, Fürsorge und vieles mehr im zwischenmenschlichen Kontext.

Bei schweren Verläufen von Coronavirus-Erkrankungen 2019 (COVID) kann es neben den bekannten die Atmung betreffenden Komplikationen auch zu erheblichen Stimm- und Schluckstörungen kommen (zum Beispiel Critical-illness-Dysphagie oder Post-Extubations-Dyphagie) [1–3].

Basierend auf intensivmedizinischen Indikationen kann es notwendig werden, eine Tracheotomie durchzuführen (unter anderem zum Schutz der oberen Atemwege oder bei fehlenden Schutzreflexen mit insuffizientem Speichelmanagement und/oder Aspiration). Dies kann eine Dysphagie zur Folge haben, da der physiologische Schluckablauf empfindlich gestört wird. Aber auch weniger invasive Eingriffe wie eine Intubation können bei längerer Liegedauer des Trachealtubus die Entwicklung einer Schluckstörung begünstigen (Post-Extubations-Dysphagie).

Je nach Krankheitsverlauf ist es das Ziel der logopädischen Therapie, die physiologische Schluckfunktion möglichst wiederherzustellen. Der erste Schritt dazu ist ein Training des Speichelmanagements (das sichere Abschlucken von Speichel ohne Aspiration). Ist dies sichergestellt, kann an weiteren Konsistenzen gearbeitet werden, um eine den aktuellen Fähigkeiten entsprechende orale Nahrungs‑, Flüssigkeits- und Medikamentenaufnahme erreichen zu können.

## Fallbericht

Eine junge Frau im Alter von 20 Jahren wurde von der Intensivstation zur logopädischen Therapie bei Schluckstörung zugewiesen. Die Zuweisungsdiagnosen lauteten Status Post-COVID-Infektion, Autismus und Intelligenzminderung. Zusätzlich bestand eine Sprachbarriere bei nichtdeutscher Muttersprache. Die Fragestellung bezog sich auf eine mögliche Oralisierung vor Übernahme von der Intensivstation auf die Erwachsenenpsychiatrie (EP).

Bei der ersten logopädischen Begutachtung befand sich die Patientin Johanna noch auf der Intensivstation. Die Patienten war vor etwa 10 Tagen extubiert worden. Es befanden sich mehrere Zugänge sowie eine Nasogastralsonde (NGS) in situ. Des Weiteren waren noch eine Sauerstoffinsufflation über eine Nasenbrille mit 4 l/min notwendig sowie mehrmaliges tägliches orales Absaugen aufgrund von oralem Speichelverlust (Drooling) und Speichelaspiration.

Beim logopädischen Erstkontakt zeigte sich Johanna somnolent und öffnete nur kurz die Augen auf Ansprache. Im gesamten Untersuchungsverlauf konnte kein einziger spontaner Schluck beobachtet werden. Dies ist als pathologisch zu werten, da eine normale Schluckfrequenz bei einem Schluck pro zwei Minuten liegt. Aufgrund der Sprachbarriere konnte ein Schlucken auf Aufforderung nicht überprüft werden. Auch manuelle Stimulationstechniken im Bereich der Kehlkopfmuskulatur konnten keinen Schluck provozieren. Somit ergab sich im standardisierten Gugging Swallowing Screen (GUSS) eine Punktanzahl von 0 [4, 5]. Dies bedeutete, dass die Patientin keinerlei Nahrung, Flüssigkeiten oder Medikamente per os zu sich nehmen durfte, da die getesteten klinischen Kriterien auf eine hochgradige Schluckstörung hinwiesen (NPO: nil per os; keinerlei orale Bolusgabe möglich).

Aufgrund der massiv reduzierten Vigilanz wurde am nächsten Tag eine erneute logopädische Evaluation mit zusätzlichem GUSS eingeplant. Johanna befand sich zu diesem Zeitpunkt bereits auf der EP. Bei diesem Kontakt war die Vigilanz deutlich gesteigert und die Patientin wendete sich auf Ansprache der Logopädin zu. Sie benötigte nach wie vor Sauerstoff über eine Maske. Die NGS wurde zum wiederholten Male in der Nacht selbstständig entfernt. Immer wieder war ein Speicheldrooling (oraler Speichelaustritt) auf der rechten Seite beobachtbar. Die Schluckfrequenz war nach wie vor deutlich herabgesetzt. Laut Pflege musste Johanna auch in der Nacht über Mund und Rachen mehrmals abgesaugt werden.

Während der therapeutischen Visitation wurde immer wieder reichlich Sekret hochgehustet und nicht abgeschluckt oder expektoriert. Es lag also weiterhin ein massives Problem mit dem Speichelmanagement vor. Der Aufforderung, willkürlich zu husten, indem man sie auf Deutsch und in ihrer Muttersprache dazu aufforderte und es ihr auch vorzeigte, konnte Johanna nicht nachkommen. Dies könnte durch die Sprachbarriere beziehungsweise durch ein reduziertes Aufgabenverständnis und/oder eine Apraxie (Unvermögen, z. B. eine Tätigkeit auszuführen) oder auch andere Vorgänge bedingt gewesen sein.

Die Schluckfunktion hat großen Einfluss auf die Lebensqualität

Die Logopädin brachte Johanna verschiedene Speisen (Obstmus, Butterbrot, Wasser) mit, um ihr Interesse am Essen wieder zu wecken, eine Essenssituation zu simulieren und dadurch die Schluckmechanismen wieder zu aktivieren.

Nach dem Psychotherapieforscher Klaus Grawe gibt es fünf zentrale Wirkfaktoren, die zu einem Therapieerfolg beitragen: Ressourcenaktivierung, Problemaktualisierung, motivationale Klärung, Problembewältigung und die therapeutische Beziehung [6]. Auch im logopädischen Therapiealltag versucht man, sich dieser Strategien, die aus der Psychotherapie kommen, zu bedienen. Im Fall von Johanna gab es sowohl eine sprachliche als auch eine kognitive Barriere, wobei man hier besonders im Bereich der Ressourcenaktivierung und Motivation im nonverbalen Bereich arbeiten musste.

Die Patientin begutachtete die Nahrungsmittel, roch auch daran, jedoch dreht sie dann jedes Mal den Kopf weg und lehnte die Angebote ab. Lediglich vom Wasser nahm sie einen kleinen Teelöffel (2–3 ml) zu sich, den sie prompt aspirierte. Der dadurch ausgelöste, reflektorische Hustenstoß war äußerst kräftig und förderte einiges an Sekret nach oben, welches dann mäßig effektiv abgeschluckt werden konnte. Es kam einerseits zu einem dyskoordinierten, mehrfachen Abschlucken, andererseits konnte man intermittierend eine klare (Glottisebene rein), dann wieder eine belegte, brodelige Stimme vernehmen (Verdacht der Speichelaspiration).

An diesem Tag erreichte der GUSS 1 Punkt für die ausreichende Vigilanz. Die klinischen Zeichen deuteten alle noch auf eine hochgradige Schluckstörung mit Aspirationsgefahr hin. Es wurde erneut eine NGS gesetzt.

Nach dem Wochenende wurde eine logopädische Reevaluation durchgeführt. Johanna hat sich die NGS zum ungezählten Male selbst entfernt, sodass sie am Wochenende parenteral ernährt wurde. Außerdem berichtete die Station, dass Johanna nicht mehr oral abgesaugt werden musste.

Sie lächelte die Therapeutin an, als diese das Zimmer betrat, und freute sich sichtlich, als sie das mitgebrachte Butterbrot und den Fruchtsaft am Tablett erblickte („therapeutische Beziehung“). Die klinisch-logopädische Begutachtung zeigte eine deutliche Besserung der schluckrelevanten Strukturen und Funktionen. Das Speicheldrooling hatte sich weitgehend zurückgebildet, die gurgelige Stimme, die auf Aspiration hindeutete, war nunmehr klar. Insgesamt schien die Patientin wacher und kräftiger von ihrer Konstitution.

Es wurden Informationen von der Familie über Vorlieben beim Essen und Trinken eingeholt. So konnte herausgefunden werden, dass Johanna bunte Nahrung bevorzugt und sehr gerne Butterbrote mit Ketchup isst („motivationale Klärung“).

Das Wissen um Vorlieben beim Essen/Trinken unterstützt die Therapie

Johanna wurde in eine gute Sitzposition gebracht und der Teller mit dem Brot vor sie hingestellt. Sie griff sogleich selbstständig zum Brot und begann es in adäquaten Bissen zu essen. Gekaut wurde wenig. Die Boli konnten jedoch gut und zunächst ohne sichtbare klinische Aspirationszeichen abgeschluckt werden. Insgesamt aß Johanna 2 ½ Butterbrote auf und trank etwas Saft.

Beim Trinken kam es zu einem geringgradigen Drooling rechts. Beim Brot verschluckte sie sich 2 × leicht, weil sie den nächsten Bissen schon genommen hatte, bevor sie komplett abgeschluckt hatte. Das Husten erfolgte unmittelbar und kräftig, und es gab keine weiteren Auffälligkeiten.

Insgesamt wirkte der Schluckablauf bei Johanna dyskoordiniert und verlangsamt. Es kam jedoch im Verlauf des Frühstücks zu keinen problematischen Situationen.

Laut Auskunft der Pflegepersonen konnte sie vor dem Ereignis nach Vorbereitung selbstständig und jede Konsistenz essen, die sie wollte. Es wurden daher einige Kleinigkeiten für das Abendessen bestellt, die ihrem Geschmack entsprechen sollten (Brot, Butter, rote Wurst et cetera).

Am nächsten Tag berichtete das Pflegepersonal, dass Johanna mit großem Appetit gegessen habe und feste Speisen bevorzugte. Gemeinsam mit der Pflege und der Diätologie wurde der weitere Speiseplan erstellt. Später teilte die Pflege der Logopädie mit, dass Johanna mittags trotz intensiver Bemühungen nichts von den angebotenen Speisen essen wollte. Schließlich wurden ihr von der Station Butterbrote mit Ketchup bereitgestellt, die sie dann mit großem Appetit verzehrte.

Am darauffolgenden Tag fand der letzte logopädische Kontrollbesuch statt. Laut Angabe der Pflegepersonen war Johanna weiterhin recht wählerisch, was ihre Ernährung betraf. Sie lehnte Speisen oft ab. Bis zuletzt präferierte sie Butterbrote mit Ketchup, die sie auch jederzeit vom Pflegepersonal bekam.

Interdisziplinäre Zusammenarbeit ist ein wichtiger Faktor in der Patient*innenbetreuung

Der letzte GUSS ergab bei weiterhin reduzierter Compliance von Johanna einen Wert von 10 Punkten. Auf Basis der klinisch-logopädischen Befunderhebung und einer Einschätzung der Aspirationsgefahr konnte jedoch eine höhere als die im GUSS bei dieser Punktanzahl beschriebene Kostform etabliert werden. Hinsichtlich der Erstellung ihres Speiseplans wurde bereits die Diätologie involviert, die sich weiter darum kümmerte. Johanna hatte keine Kostformeinschränkung mehr, sodass aktuell seitens der Logopädie keine weitere Notwendigkeit einer Observanz bestand.

Im Laufe der Besuche bei Johanna war zu beobachten, dass sich das Vertrauen in Einzelpersonen bzw. in das Stationspersonal veränderte. Zunächst lehnte Johanna vermehrt die Angebote von Speisen und Getränken ab. Sie ließ sich jedoch zunehmend auf ihre Umgebung ein.

Im Stationsalltag wurde Johanna offener, musste aber auch aufgrund ihrer Verhaltensauffälligkeiten 1:1 betreut werden, um heikle Situationen zu vermeiden.

Das Pflegepersonal ging speziell auf ihre Bedürfnisse ein, sodass Johanna sich immer sicherer zu fühlen schien und die Nähe zum Personal suchte.

## Fazit für die Praxis

- Eine regelmäßige Schluckevaluierung determiniert die Anpassungsmöglichkeiten für die Ernährung, Flüssigkeits- und Medikamentengabe.
- Die Aspirationsgefahr kann mithilfe einer logopädischen Schluckevaluierung unter Verwendung eines standardisierten, evidenzbasierten Instruments wie z. B. dem GUSS eingeschätzt werden.
- Die schrittweise Anpassung der Kostform baut auf den vorgenannten Punkten auf. Ziel ist immer eine den Möglichkeiten entsprechende sichere aspirationsfreie orale Bolusaufnahme.
- Die genaue Auseinandersetzung mit der Anamnese vor und im Behandlungszeitraum liefert wichtige Hinweise für die Planung und Durchführung der Therapie.
- Die Einbeziehung aller Berufsgruppen, die für die Patient*innen erforderlich sind, (Ärzt*innen, Pflegepersonal, Logopädie, Physiotherapie, Diätologie) ist unerlässlich.
- Die Kooperation mit der Familie sollte in jedem Fall gesucht werden, um entsprechend auf die Patient*innen eingehen zu können.
